# Three hypomethylated genes were associated with poor overall survival in pancreatic cancer patients

**DOI:** 10.18632/aging.101785

**Published:** 2019-02-01

**Authors:** Huiming Chen, Yan Kong, Qing Yao, Xing Zhang, Yunong Fu, Jia Li, Chang Liu, Zheng Wang

**Affiliations:** 1 Department of Hepatobiliary Surgery, The First Affiliated Hospital of Xi’an Jiaotong University, Xi’an 710061, Shaanxi, China; 2 Department of General Surgery, Shaanxi Provincial Rehabilitation Hospital, Xi’an 710065, Shaanxi, China; 3 Department of Clinical Laboratory, Liaocheng People’s Hospital, Taishan Medical College, Liaocheng 252000, Shandong, China; 4 Department of Orthopaedics, The First Affiliated Hospital of Xi’an Jiaotong University, Xi’an 710061, Shaanxi, China

**Keywords:** pancreatic cancer, DNA methylation, SULT1E1, IGF2BP3, MAP4K4, prognostic model

## Abstract

Pancreatic cancer (PC) is a highly malignant cancer with poor prognosis and high mortality. Aberrant DNA methylation plays a critical role in the occurrence, progression and prognosis of malignant tumors. In this study, we employed multiple datasets from APGI, TCGA and GEO to perform Multi-Omics analysis, including DNA methylation and expression profiling analysis. Three differentially expressed genes (SULT1E1, IGF2BP3, MAP4K4) with altered status of DNA methylation were identified and then enrolled into prognostic risk score model using LASSO regression. Univariate cox regression analysis indicated that high risk score was significantly associated with poor prognosis. Multivariate cox regression analysis proved the risk score was an independent prognostic factor for PC. In addition, time-dependent ROC curves indicated good performance of our model in predicting the 1-, 3- and 5-year survival of PC patients. Besides, stratified survival analysis revealed that the risk score model had greater prognostic value for patients of late stage with T3/T4 and N+. Pathway enrichment analysis suggested that these three genes might promote tumor progression by affecting signaling by Rho GTPases and chromosome segregation. In summary, three hypomethylated gene signature were significantly associated with patients’ overall survival, which might serve as potential prognostic biomarkers for PC patients.

## Introduction

Pancreatic cancer (PC) is a highly malignant cancer with poor prognosis and high mortality which almost parallels to its disease incidence. It is reported that PC has become the fourth leading cause of cancer-related death in the USA and are predicted to be the second leading cause of cancer death in the USA by 2030 [1]. What’s worse, most patients with PC are asymptomatic at early stage. Therefore, it is quite often that PC patients have reached late stage when they are diagnosed, leading to the poor prognosis [2]. Aside from the progress in surgical resection and other adjuvant therapies, exploring efficient methods of early diagnosis is another important way to improve the clinical prognosis of PC patients. Compared to CT scanning and MRI, the more specific and sensitive biomarkers present greater value in early diagnosis, prognosis prediction and even therapeutic treatment.

DNA methylation, as one of the major epigenetic modifications, plays an important role in the occurrence and progression of malignant tumors [3]. Aberrant DNA methylation of CpG islands located in promoter regions, as a critical molecular mechanism of tumor occurrence, often leads to transcriptional silence of tumor suppressor genes and over-expression of oncogenes through hypermethylation and hypomethylation respectively [4]. Notably, increasing studies have identified a series of gene mutations with deregulated DNA methylation from PC tissues [5]. For instance, KRAS (Kirsten rat sarcoma viral oncogene homolog) was found to be the most frequently mutated oncogene in PCs which involves in cellular proliferation, motility, and cytoskeletal remodeling in the earliest stage of pancreatic tumorigenesis [6]. And targeting oncogenic KRAS using exosomes was reported to significantly increase overall survival in PC mice models [7]. Therefore, it is of promising value for diagnosis and treatment of PC patients to identify specific genes with aberrant DNA methylation in PC.

Although the association between DNA methylation and prognosis of PC has been extensively reported, specific prognostic model was rarely reported. In this study, by using the combination of methylation and expression profiling data, we aimed to identify the prognostic significance of differentially expressed genes (DEGs) with altered DNA methylation status in PC and to set up a reliable prognostic model for PC patients.

## RESULTS

### Identification of DEGs with altered DNA methylation status in PC

**Figure 1:**
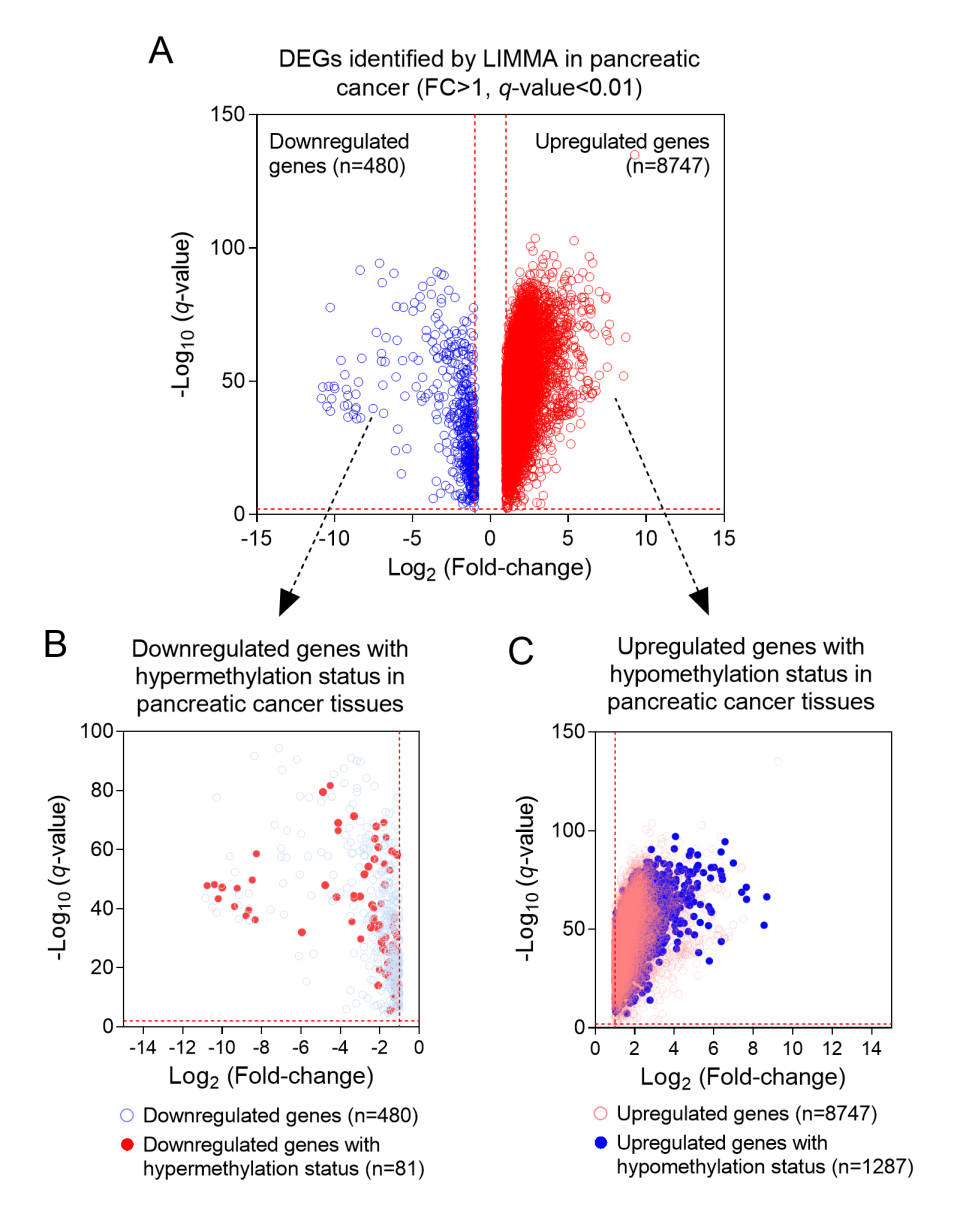
**The process of screening candidate genes. (A**) The DEGs in PC and normal tissues (n=9227); (**B**) Downregulated genes with hypermethylation status (n=81); (**C**) Upregulated genes with hypomethylation status (n=1287).

**Figure 2:**
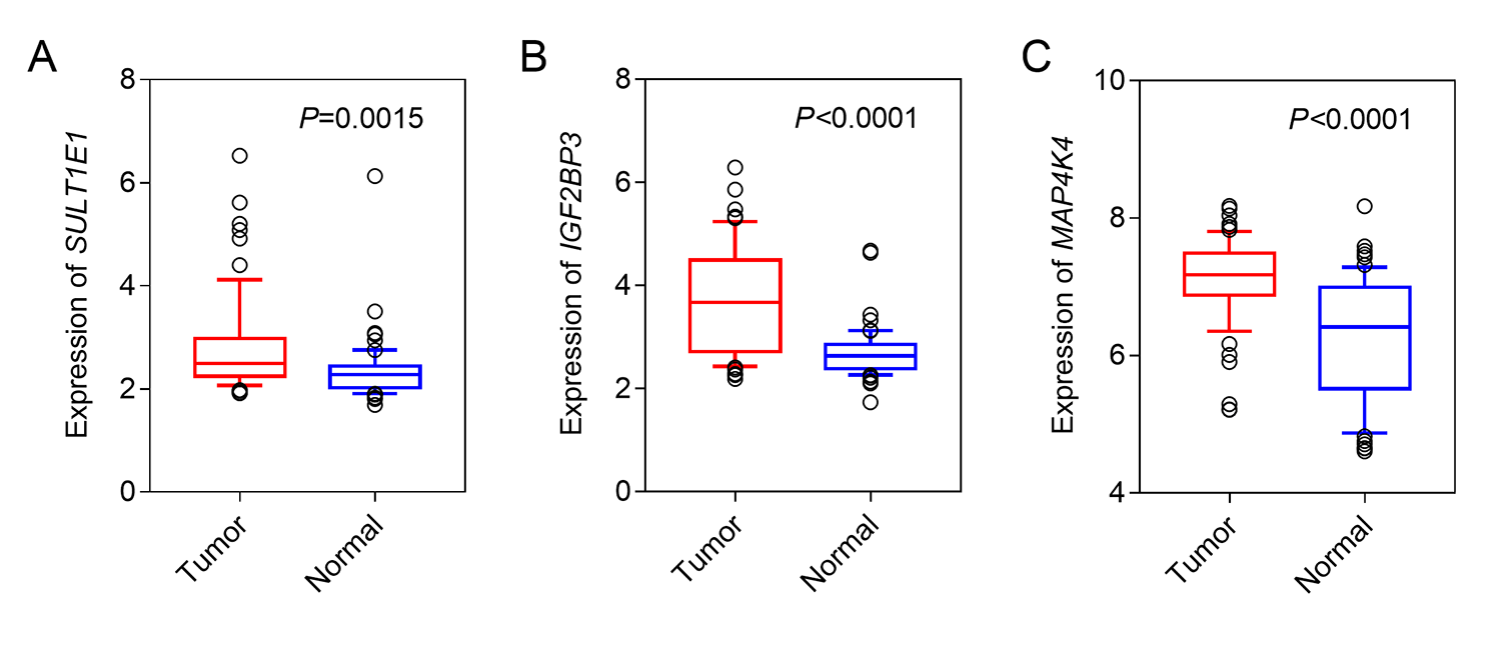
**The expression of three hypomethylated genes in PC and normal tissues.** (**A**) SULT1E1; (**B**) IGF2BP3; (**C**) MAP4K4.

We firstly conducted LIMMA analysis to select DEGs using expression data from GSE62452 dataset. A total of 9227 DEGs, including 480 down-regulated genes and 8747 up-regulated genes were identified (Foldchange>1, *q*-value<0.01) (Figure 1A). By comparing with the DNA methylation patterns of the ICGC dataset, we further identified 81 down-regulated genes which were hypermethylated (81/480, 16.9%) (Figure 1B) and 1287 up-regulated genes which were hypomethylated (1287/8747, 14.7%) (Figure 1C). Next, by performing univariate cox regression analysis between 1368 candidate genes above and survival data of discovery cohort, we obtained 3 prognosis-related genes SULT1E1, IGF2BP3 and MAP4K4, which all reached a statistical significance (P<0.05). The differential expression of above three genes between tumor and normal tissues was further validated in an independent cohort, GSE62452, which contained a total of 69 tumor and 61 normal tissues. Our results revealed that all three genes (SULT1E1, IGF2BP3 and MAP4K4) were all significantly overexpressed in PC tissues, suggesting that they might be potential biomarkers for PC patients (Figure 2).

### Construction and assessment of prognostic risk score model for PC

**Figure 3:**
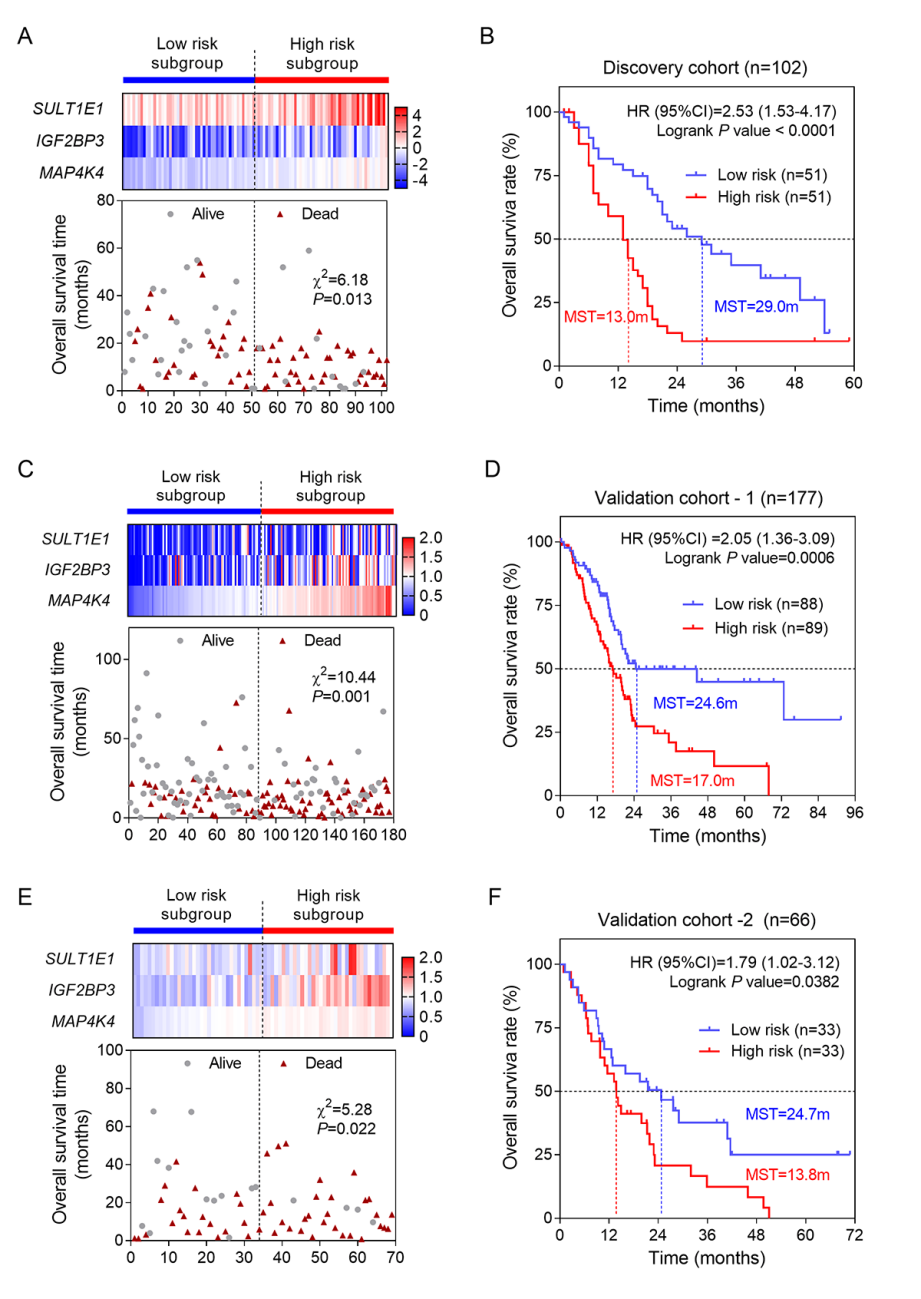
**Construction and validation of three-gene risk score model.** (**A**) The heatmap and distribution of the three gene expression profiles in the high-risk and low-risk subgroups for the discovery cohort; (**B**) Kaplan-Meier analysis of patients’ OS in the high-risk and low-risk subgroups of the discovery cohort; (**C**) The heatmap and distribution of the three gene expression profiles for the validation-1 cohort; (**D**) Kaplan-Meier analysis of the validation-1 cohort; (**E**) The heatmap and distribution of the three gene expression profiles for the validation-2 cohort; (**F**) Kaplan-Meier analysis of the validation-2 cohort.

We selected these three genes to conduct risk score model for PC patients using LASSO regression method. Our risk score formula obtained from discovery cohort was that risk score=0.195 * expression of SULT1E1+ 0.129 * expression of IGF2BP3 + 0.65 * expression of MAP4K4. According to the formula, we calculated the risk score for each patient. Then patients of each cohort were divided into high- and low-risk subgroups by the median value in order to evaluate the prognostic value of risk score by performing univariate and multivariate OS analysis. The results were shown in Table 1. Remarkably, the consistent results of three cohorts (discovery and two validation cohorts) evidently prove that risk score was associated with OS. After adjusted by using AJCC stage and histological grade, our results also reached a statistically significant, suggesting that risk score was an independent prognostic factor for PC. The patients in high risk score group had a two-fold higher risk of death than those in low risk score group (Table 1 and Figure 3A, 3C and 3E). Moreover, we conducted Kaplan-Meier curves to evaluate the impact of risk score on patients’ OS time. Our results showed that patients with high risk score had significantly poorer OS than patients with low risk score in three cohorts (Figure 3B, 3D and 3F). The median survival time (MST) of patients with high risk score was less than 1.5 years (ranged from 13.0-17.0m) while themedian MST of patients with low risk score was more than 2.0 years (ranged from 24.6-29.0m).

**Table 1:** Univariate and multivariate survival analysis of three cohorts.

Variable	Discovery cohort					Validation-1 cohort					Validation-2 cohort				
	Univariate analysis			Multivariate analysis		Univariate analysis			Multivariate analysis		Univariate analysis			Multivariate analysis	
	N	*P*-value	HR(95%CI)	*P*-value ^a^	HR(95%CI) ^a^	N	*P*-value	HR(95%CI)	*P*-value ^b^	HR(95%CI) ^b^	N	*P*-value	HR(95%CI)	*P*-value ^c^	HR(95%CI) ^c^
**AJCC stage**															
I/IIA	26		Ref		Ref	48		Ref		Ref	14		Ref		Ref
IIB/III/IV	71	**0.038**	1.79(1.07-3.00)	0.138	1.57(0.86-2.86)	124	**0.013**	1.89(1.22-2.93)	0.908	1.07(0.33-3.51)	52	0.125	1.69(0.92-3.09)	0.064	3.36(0.93-12.13)
**T stage**															
T1/T2	18		Ref		Ref	31		Ref		Ref	NA				
T3/T4	80	0.863	0.95(0.51-1.76)	0.209	1.55(0.78-3.06)	143	**0.022**	2.04(1.24-3.34)	0.413	1.34(0.67-2.68)	NA	NA	NA	NA	NA
**N stage**															
N0	28		Ref		Ref	48		Ref		Ref	20		Ref		Ref
N+	73	**0.027**	1.80(1.09-2.95)	0.138	1.57(0.86-2.86)	123	**0.006**	2.03(1.31-3.15)	0.402	1.63(0.52-5.15)	46	0.296	1.38(0.77-2.47)	0.564	0.73(0.26-2.10)
**Histological grade**															
G1/G2	NA					125		Ref		Ref	34		Ref		Ref
G3/G4	NA	NA	NA	NA	NA	50	0.064	1.50(0.94-2.40)	0.339	1.24(0.80-1.93)	31	**0.018**	1.87(1.05-3.32)	0.272	3.25(0.40-26.69)
**Risk score**															
low risk	50		Ref		Ref	88		Ref		Ref	33		Ref		Ref
high risk	51	**<0.0001**	2.53(1.53-4.17)	**0.022**	1.82(1.09-3.05)	89	**0.0006**	2.05(1.36-3.09)	**0.023**	1.67(1.07-2.60)	33	**0.038**	1.79(1.02-3.12)	**0.009**	2.29(1.23-4.24)

Abbreviation: N, number; HR, hazard ratio; CI, confidence interval; Ref, reference; AJCC, American Joint Committee on Cancer; NA, not available.

^a^ multivariate analysis was adjusted by AJCC stage, T stage, N stage

^b^ multivariate analysis was adjusted by AJCC stage, T stage, N stage, Histological grade

^c^ multivariate analysis was adjusted by AJCC stage, N stage, Histological grade

### Stratified survival analysis

**Figure 4:**
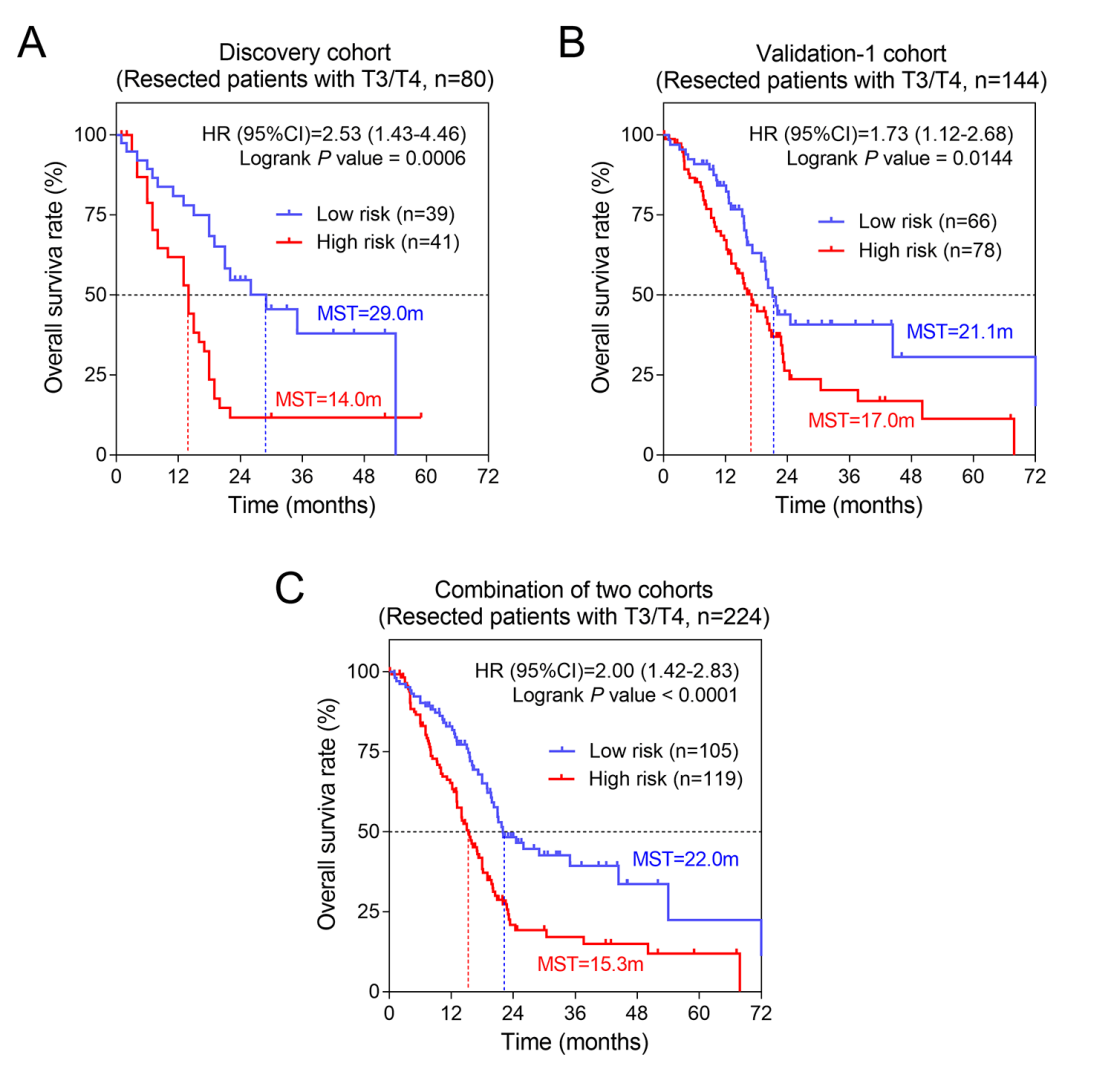
**The three-gene signature was associated with prognosis in patients with advanced stage.** Kaplan-Meier analysis of the OS of patients with advanced stage in discovery cohort (**A**) and validation-1 cohort (**B**). (**C**) Kaplan-Meier analysis was performed by combining of above two cohorts.

**Figure 5:**
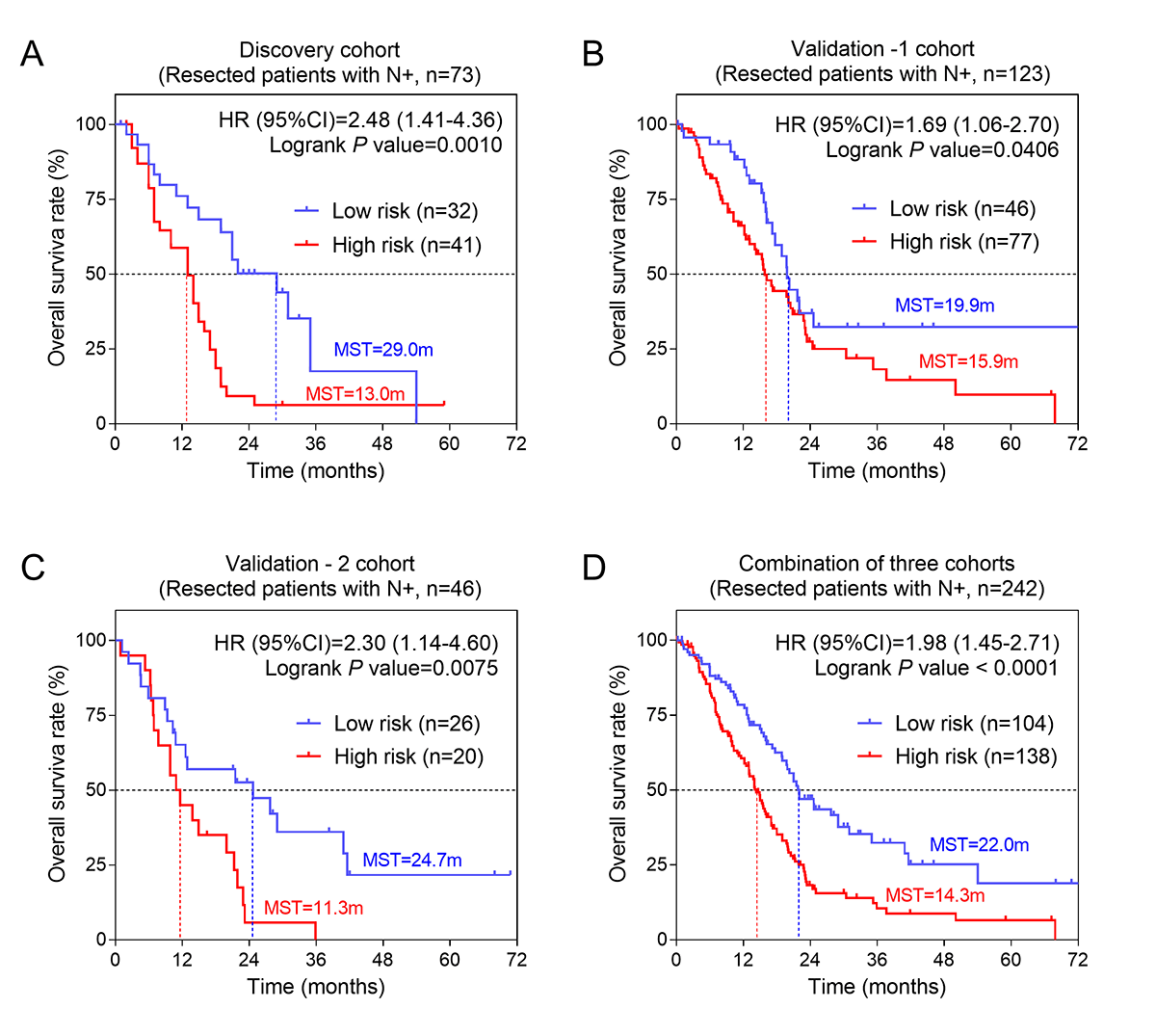
**The three-gene signature was associated with prognosis in patients with metastatic lymph nodes.** Kaplan-Meier analysis of the OS of patients with metastatic lymph nodes in discovery cohort (**A**), validation-1 cohort (**B**) and validation-2 cohort (**C**). (**D**) Kaplan-Meier analysis was performed by combining of above three cohorts.

The univariate analysis showed that AJCC stage, T, N and histological grade had relatively significant impact on prognosis (Table 1). Therefore, we further performed stratified survival analysis to evaluate the prognostic values of our risk score model in different subgroups. According to the results of Table 2, in general, for patients of advanced stage with T3/T4 stage and metastatic lymph nodes (N+), risk score revealed greater prognostic value. Specifically, for T3/T4 subgroup, results remained consistently significant in discovery cohort (P= 0.0006, HR= 2.53, 95%CI: 1.43-4.46) and validation-1 cohort (P= 0.0144, HR= 1.73, 95%CI: 1.12-2.68) (Figure 4). Similarly, we also found the significant prognostic values of risk score in PC patients with N+. The results for N+ subgroup in three cohorts were showed in Figure 5. Especially, for validation-1 cohort, when stratified by T or N, the results of subgroups remained stable, which, to some extent, indicated the reliability and general applicability of our risk score model.

**Table 2:** Stratified survival analysis according to major clinical factors of three cohorts.

Variable	Discovery cohort				Validation-1 cohort				Validation-2 cohort				
	Low risk	High risk	*P*-value	HR(95%CI)	Low risk	High risk	*P*-value	HR(95%CI)	Low risk	High risk	*P*-value		HR(95%CI)
**AJCC stage**													
I/IIA	16	10	0.131	2.09(0.69-6.33)	32	17	**0.026**	2.66(0.94-7.51)	4	10		0.125	4.33(1.15-16.27)
IIB/III/IV	30	41	**0.001**	2.41(1.36-4.27)	53	72	0.064	1.57(0.99-2.48)	29	23		**0.020**	2.00(1.04-3.85)
**T stage**													
T1/T2	9	9	0.086	2.31(0.73-7.32)	19	12	**0.028**	3.58(1.02-12.59)	NA	NA		NA	NA
T3/T4	39	41	**0.0006**	2.53(1.43-4.46)	66	78	**0.014**	1.73(1.12-2.68)	NA	NA		NA	NA
**N stage**													
N-	18	10	0.135	2.01(0.68-5.95)	30	19	**0.017**	2.86(1.05-7.81)	7	13		0.467	1.53(0.52-4.50)
N+	32	41	**0.001**	2.48(1.41-4.36)	46	77	**0.041**	1.69(1.06-2.70)	26	20		**0.008**	2.30(1.14-4.60)
**Histological grade**													
G1/G2	NA	NA	NA	NA	66	59	**0.0003**	2.53(1.51-4.24)	25	9		0.674	1.20(0.50-2.88)
G3/G4	NA	NA	NA	NA	19	31	0.447	1.33(0.65-2.71)	7	24		0.399	1.50(0.63-3.60)

Abbreviation: HR, hazard ratio; CI, confidence interval; AJCC, American Joint Committee on Cancer; NA, not available

### A comparison between our and other models

**Figure 6:**
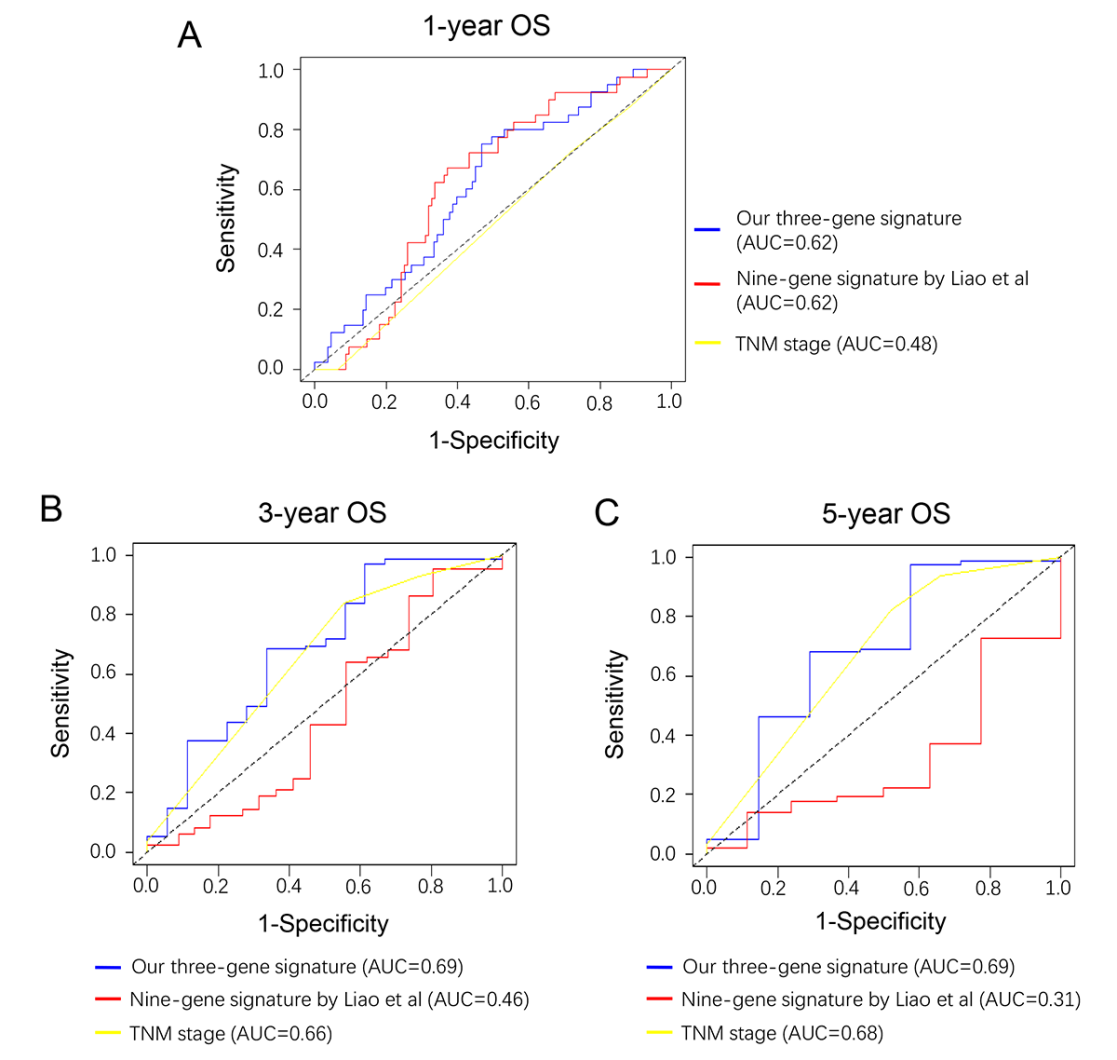
**Comparison of our three-gene model and other literature models.** Time-dependent ROC analysis was performed to compare the three models in predicting 1-year (**A**), 3-year (**B**) and 5-year (**C**) OS.

Recently, Liao et al reported a model containing 9 genes based on TCGA cohort [8]. To compare the prognostic values of our three-gene model and their model, we performed time-dependent ROC curve analysis. The results showed that the Liao’s model exhibited a favorable predictive value in predicting 1-year OS, however, its predictive value obviously decreased in predicting 3- and 5- years OS. By contrast, the predictive values of AJCC stage, which was considered as recommended model, showed a better efficiency in predicting 3- and 5-years OS, but failed to predict 1-year OS. The area under ROC curve (AUC) of our three-gene model for 1-, 3- and 5-year overall survival was 0.62, 0.69, and 0.69, respectively, suggesting our three-gene model had a favorable efficiency in predicting both short- and long-term prognosis (Figure 6).

### Biological function prediction

**Figure 7:**
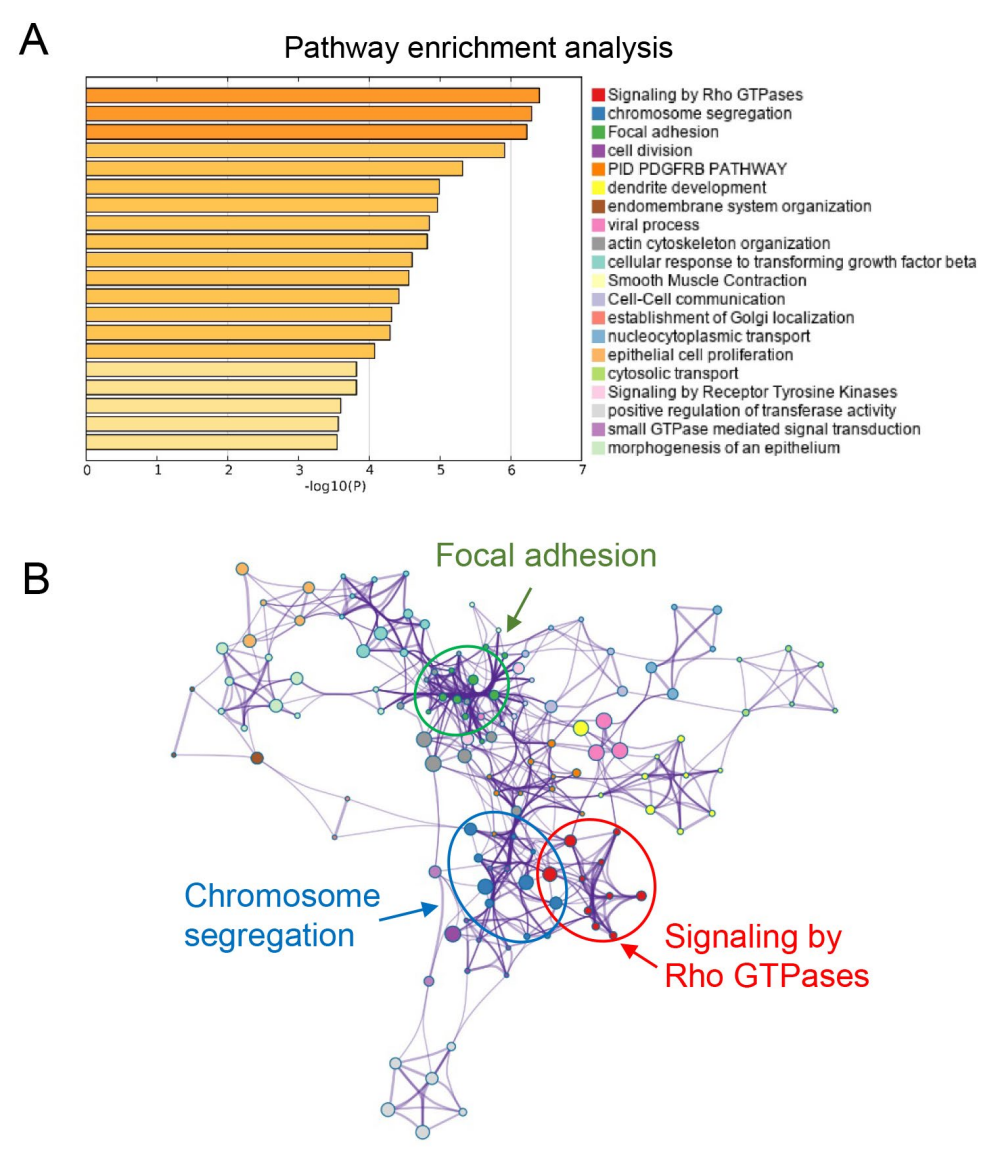
**Functional prediction of three-gene model.** (**A**) Significantly enriched pathways of the three genes and their co-expressed genes. (**B**) The functional enrichment map of pathways. Each node represents a GO term. Node size represents the number of gene in the pathways.

To explore the potential biological function of the three genes, we performed pathway enrichment analysis. Our data suggested that the top three signaling pathways that affected by SULT1E1, IGF2BP3, MAP4K4 and their co-expressed genes were Rho GTPases, chromosome segregation and focal adhesion pathways (Figure 7). Above three signaling pathways were all reported to be involved in tumor progression, providing evidence for further investigating the detailed molecular mechanisms of our three-gene models in PC.

## DISCUSSION

Lack of effective and reliable prognostic biomarkers or models remains as a major problem for improving the clinical outcomes of PC patients. In this paper, we aim to explore and evaluate the prognostic values of methylated genes for PC. In brief, we firstly obtained 1368 DEGs with altered DNA methylation status. Three out of them (SULT1E1, IGF2BP3, MAP4K4) were identified as prognosis-related genes and were selected to generate a risk score model. Survival analysis proved our three-gene model was an independent prognostic factor for PC. Stratified analysis further revealed that the risk score model had a greater prognostic value for patients of advanced stage and metastatic lymph nodes. In conclusion, our three-gene model might serve as a potential predictive tool for PC patients.

During the past decades, a number of studies exploring the prognostic models for PC patients were reported. In the present study, we conducted a three-gene risk model based on DNA methylation and gene expression profiling datasets. Time-dependent ROC curve showed that our three-gene model exhibited a stable and good performance in prognosis prediction. Our model shows good accuracy and stability in clinical outcome prediction either for 1-year (AUC=0.62), 3-year (AUC=0.69) and 5-year (AUC=0.69) OS of PC patients, when compared with traditional AJCC stage. Recently, Liao et al reported a nine-gene prognostic model [8] based on early stage patients (stage I & II). The accuracy of our three-gene model for predicting 1-year survival was comparable to Liao’s with both AUC=0.62. However, when predicting long-term (3- and 5- year) survival, Liao’s nine-gene model showed relatively poor performance. Meanwhile, our model exhibited an increased AUC and indicated a better prediction efficacy for predicting 3- and 5-year survival, which is relatively more important for patients of advanced stage. Consistent with the better prognostic value for patients with T3/T4 and N+, it can be concluded that our prognostic model had great significance for predicting long-term survival of advanced PC patients.

All the three genes (SULT1E1, IGF2BP3 and MAP4K4) in our model were confirmed to be upregulated in PC tissues. Our data also revealed that the three-gene model was associated with poor prognosis, especially in patients with advanced stage. Among them, MAP4K4, a serine/threonine kinase involved in activation of the JNK signaling pathway, was overexpressed in many types of human cancer and played an important role in proliferation, migration and invasiveness of cancer cells [9]. It was reported that the expression levels of MAP4K4 in CRC patients with lymph node metastasis were higher than that in patients without metastasis [10]. IGF2BP3 was initially identified as an oncofetal gene due to its high abundance in PC tissue and proved relevant with aggressive and invasive phenotype. IGF2BP3 was found to be upregulated in a variety of malignant tumors including lung, esophageal cancers and melanomas, and was capable of promoting tumor growth, drug-resistance and metastasis. Besides, expression of IGF2BP3 was reported to be associated with poor prognosis and metastasis [11]. SULT1E1 was a member of SULT1 family and is best known for inactivating estrogen in humans [12]. SULT1E1 was found to be correlated with estrogen-dependent breast and endometrial cancer while the expression level remains controversial [13]. High SULT1E1 levels were found in breast cancer tissues and associated with a poor prognosis for breast cancer in women [14]. Experimental data showed that SULT1E1 overexpression inhibited proliferation, migration, invasion of breast cancer cells by mediating the adaptive response to estrogen in tumor cells [15]. Different from the above two genes, the role of SULT1E1 in PC was rarely reported and remains obscure. Recently, Seeliger et al reported that estrogen receptor expression was an independent predictor of shorter OS in resected PC patients [16]. Our findings therefore provide insight into the underlying mechanisms of estrogen-related signaling in PC progression.

To date, there was limited prognostic model for patients with metastatic lymph nodes. In this study, we also found that the prognostic efficiency of three-gene model was increased in patients with metastatic lymph nodes. Liang et al reported that MAP4K4 overexpression was associated with increased number of metastatic lymph nodes [17]. Yang et al also found that the expression of SULT1E1 was significantly higher in PC tissues with lymph nodes metastasis than in PC tissues without lymph nodes metastasis [18]. Yet, there was no direct evidence for the association between IGF2BP3 and lymph nodes metastasis in PC. Nevertheless, an experiment performed by Satoru et al showed that the H19-PEG10/IGF2BP3 axis promotes the progression of high lymph node ratio (the ratio of the number of metastatic lymph nodes to the number of dissected lymph nodes) in gastric cancer [19]. Above evidence were consistent with and confirmed our findings of the association between three-gene model and lymph node metastasis.

Several limitations in our study should be pointed out. First, because the clinical information of patients was limited, we could not perform subgroup analysis by stratifying more factors. Second, the censored rate of validation-1 cohort was high, which may comprise the reliability of the Kaplan-Meier estimates. Third, the construction and assessment of this prognostic model was based on public datasets. To further confirm or refuse this model, we warrant large-size, multicenter and prospective clinical cohorts in future.

In summary, we identified 1368 differentially expressed genes with altered DNA methylation and selected three of them (SULT1E1, IGF2BP3 and MAP4K4) to construct a prognostic model. Survival analysis showed that our risk score model exhibited significant prognostic value for PC patients, especially for patients with advanced stage and metastatic lymph node. By comparing with AJCC stage and other models from literature, our model presented greater advantages in stability and accuracy for prognosis prediction and was promising to be applied for clinical prognostic evaluation of PC patients.

## MATERIALS AND METHODS

### Data and sources

The set of array-based DNA methylation data of PC were obtained from the Australian PC Genome Initiative (APGI; https://www.garvan.org.au/research/cancer/pancreatic-cancer-research/). The set of sequence-based mRNA expression data (RNA-seq data) of PC was downloaded from The Cancer Genome Atlas (TCGA) (https://cancergenome.nih.gov/). Another two gene expression arrays of human PC datasets (GSE21501 and GSE62452) were obtained from the Gene Expression Omnibus (GEO) (https://www.ncbi.nlm.nih.gov/gds/) and were served as the discovery cohort (n=102) and validation cohort (n=66), respectively. Moreover, clinicopathological information and survival data of total 345 PC patients from three cohorts (TCGA, GSE21501 and GSE62452) were also obtained for further prognostic analysis.

### Identification of DEGs with altered methylation status in PC

**Figure 8:**
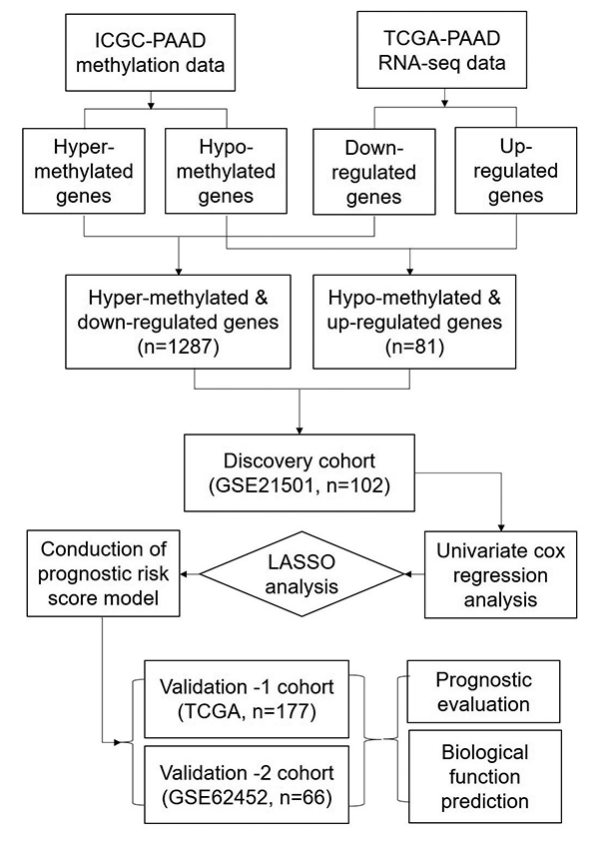
The workflow of construction and evaluation of our prognostic model.

The expression dataset and DNA methylation dataset were employed to identify the differentially expressed genes with altered methylation status. The flowchart of this study is shown in Figure 8. Firstly, LIMMA analysis was performed to identify the DEGs by comparing the normalized expression data between PC and adjacent normal tissues. We used GSE62452 to validate the differentially expressed genes because it consisted of 69 tumor tissues and 61 normal tissues. Next, we compared DNA methylation status of genes from the ICGC dataset and divided them into hyper-methylated genes and hypo-methylated genes. Then we correlated the level of RNA expression with the degree of DNA methylation and in order to classify genes into two groups, hyper-methylated & down-regulated group and hypo-methylated & up-regulated group. Genes of both groups were selected as candidate genes.

### Establishment of prognostic model and receiver operating characteristic (ROC) curve

A univariate Cox regression analysis was firstly performed on all of candidate genes in discovery group to calculate the association between the expression level of each gene and patient's overall survival (OS). Those genes with P-values less than 0.05 were identified as prognosis-related genes (key genes). Then, the selected key genes were further screened and confirmed by the Lasso regression. The prognosis risk score was established with the following formula: Risk score = expression of Gene 1 * β1 + expression of Gene 2 * β2 +…expression of Gene n * βn. In this case, we would be able to generate a risk score for each patient of 3 cohorts based on the normalized expression data. Patients were then divided into high- and low- risk subgroups according to the median cutoff of the prognosis risk score. The prognostic performance was evaluated by using time-dependent receiver operating characteristic (ROC) curve analysis within 1 year, 3 years and 5 years to evaluate the predictive accuracy and sensitivity of our prognostic model.

### Overall and stratified survival analysis

Survival data were presented as frequency and percentage both for categorical variables and continuous variables (converted into categorical variables). Univariate cox regression survival analysis was firstly performed to evaluate the prognostic effect of risk score and various clinicopathological features including age, gender, tumor stage, grade, history of chronic pancreatitis, history of alcohol, history of diabetes, family history of cancer. Then the ones of prognostic significance would be put into a cox proportional hazards model for multivariate cox regression survival analysis to further validate if risk score was an independent factor for prognosis. In addition, to further explore the influence of other factors on the prognostic value of risk score, stratified analysis was performed according to prognosis-related clinical features including stage (I/IIA and IIB/III/IV), T stage (T1/T2 and T3/T4), N (N- and N+) and histological grade (G1/G2 and G3/G4). The log-rank test was chosen to determine significant differences of survival curves. A two-sided P value< 0.05 was considered statistically significant. Hazard radio (HR) and 95% confidence interval (CI) were reported if necessary. Statistical analysis was performed using IBM SPSS version 24.0 (IBM Corp, NY, USA). Kaplan-Meier survival was curved by Graphpad prism 7.

### Pathway enrichment analysis

The enrichment analysis was conducted to predict the biological function of the three genes. We calculated the Pearson’s correlation coefficients between each of three hypomethylated genes and genome and selected the most strongly co-expressed genes (top 200) for each hypomethylated gene. Then we performed pathway enrichment analysis using above genes. A web-based tool, Metascape (http://metascape.org/), was employed to gain insights into the biological functions of these co-expressed genes.
